# A novel *HECW2* variant in an infant with congenital long QT syndrome

**DOI:** 10.1038/s41439-023-00245-w

**Published:** 2023-06-06

**Authors:** Rina Imanishi, Kouichi Nakau, Sorachi Shimada, Hideharu Oka, Ryo Takeguchi, Ryosuke Tanaka, Tatsutoshi Sugiyama, Mitsumaro Nii, Toshio Okamoto, Ken Nagaya, Yoshio Makita, Kumiko Yanagi, Tadashi Kaname, Satoru Takahashi

**Affiliations:** 1 Department of Pediatrics, Asahikawa Medical University, Hokkaido, Japan; 2 Division of Neonatology, Center for Maternity and Infant Care, Asahikawa Medical University Hospital, Hokkaido, Japan; 3 Department of Genetic Counseling, Asahikawa Medical University Hospital, Hokkaido, Japan; 4 Department of Genome Medicine, National Center for Child Health and Development, Tokyo, Japan

**Keywords:** Disease genetics, Neurodevelopmental disorders

## Abstract

Pathogenic variants of *HECW2* have been reported in cases of neurodevelopmental disorder with hypotonia, seizures, and absent language (NDHSAL; OMIM #617268). A novel *HECW2* variant (NM_001348768.2:c.4343 T > C,p.Leu1448Ser) was identified in an NDHSAL infant with severe cardiac comorbidities. The patient presented with fetal tachyarrhythmia and hydrops and was postnatally diagnosed with long QT syndrome. This study provides evidence that *HECW2* pathogenic variants can cause long QT syndrome along with neurodevelopmental disorders.

Congenital long QT syndrome (LQTS) is a genetic disorder characterized by propensity for life-threatening cardiac arrhythmias that affects 1 in 2000 infants^1^. It is caused by pathogenic variants most commonly involving potassium and sodium ion channels and can be inherited or caused by a de novo variant^2^. To date, pathogenic variants in 17 different genes have been reported in LQTS; however, over 90% of variant-positive LQTS cases can be accounted for by three major LQTS genes—*KCNQ1*, *KCNH2*, and *SCN5A*^2^. A causal variant is found in approximately 75% of patients with LQTS with a definite phenotype; however, the genetic background of the remaining 25% of patients is unknown^3^. Here, using whole exome sequencing and variant filtering analysis, we identified a de novo heterozygous missense variant in *HECW2* in an infant with congenital long QT syndrome who had no pathogenic variant in the known LQTS-associated genes. *HECW2* encodes an E3 ubiquitin-protein ligase that stabilizes and enhances the transcriptional activity of p73, a key regulator of cell proliferation and apoptosis^4^. The encoded protein is highly expressed in the brain, lung, and heart tissue^4^. Recently, *HECW2* variants have been reported to cause neurodevelopmental disorder with hypotonia, seizures, and absent language (NDHSAL; OMIM #617268). Of the 37 cases of NDHSAL reported to date, only 4 had cardiac comorbidities (Supplementary Table 1)^5,6^. This study reports an additional case of *HECW2*-related disorder with cardiac involvement and provides further evidence that *HECW2* pathogenic variants can cause LQTS along with neurodevelopmental disorders.

The patient, now aged 2 years, is the first child of healthy, non-consanguineous Japanese parents. The mother was referred at 26 weeks of gestation for fetal echocardiography as tachycardia was discovered during an incidental ultrasound examination. M-mode echocardiography revealed intermittent tachycardia with a heart rate of 190 beats per minute (Fig. 1). Supraventricular tachycardia was suspected, and the fetal heart rate was closely monitored. Ultrasound examination at 27 weeks of gestation revealed fetal hydrops, most likely resulting from tachycardia. After confirming that the maternal electrocardiogram (ECG) was normal, trans-maternal treatment was initiated with digoxin followed by sotalol. However, the fetus remained in intermittent tachycardia. A 1689 g female neonate was delivered by cesarean section at 33 weeks of gestation due to maternal drug intolerance. A postnatal ECG demonstrated prolongation of the corrected QT interval (QTc [Fridericia], 682 msec) and torsade de pointes (Fig. 2A), which led to a diagnosis of congenital LQTS.

**Fig. 1:**
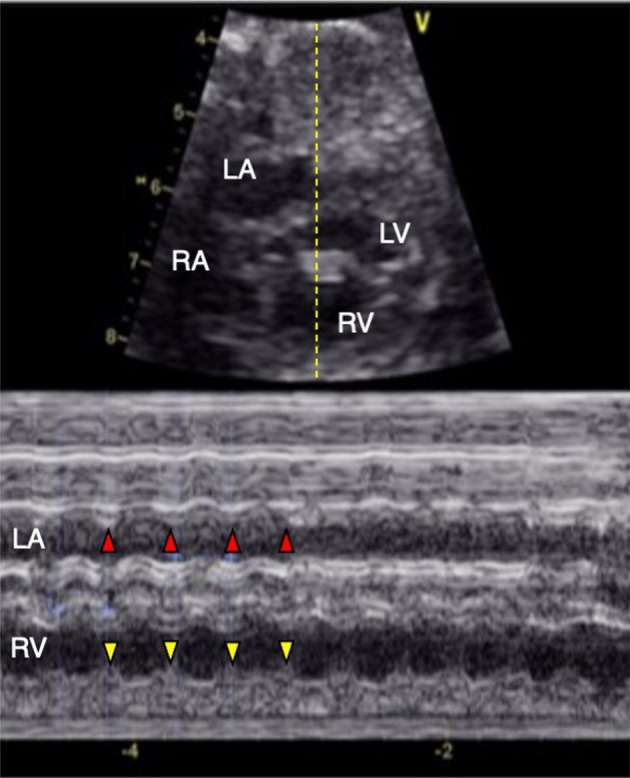
Fetal echocardiogram. 2D mode (upper) shows the ultrasound line used for M-mode (lower) echocardiogram crossing the left atrium (LA) and the right ventricle (RV). M-mode shows tachycardia with a heart rate at 190 beats per minute. LA left atrium, LV left ventricle, RA right atrium, RV right ventricle.

**Fig. 2:**
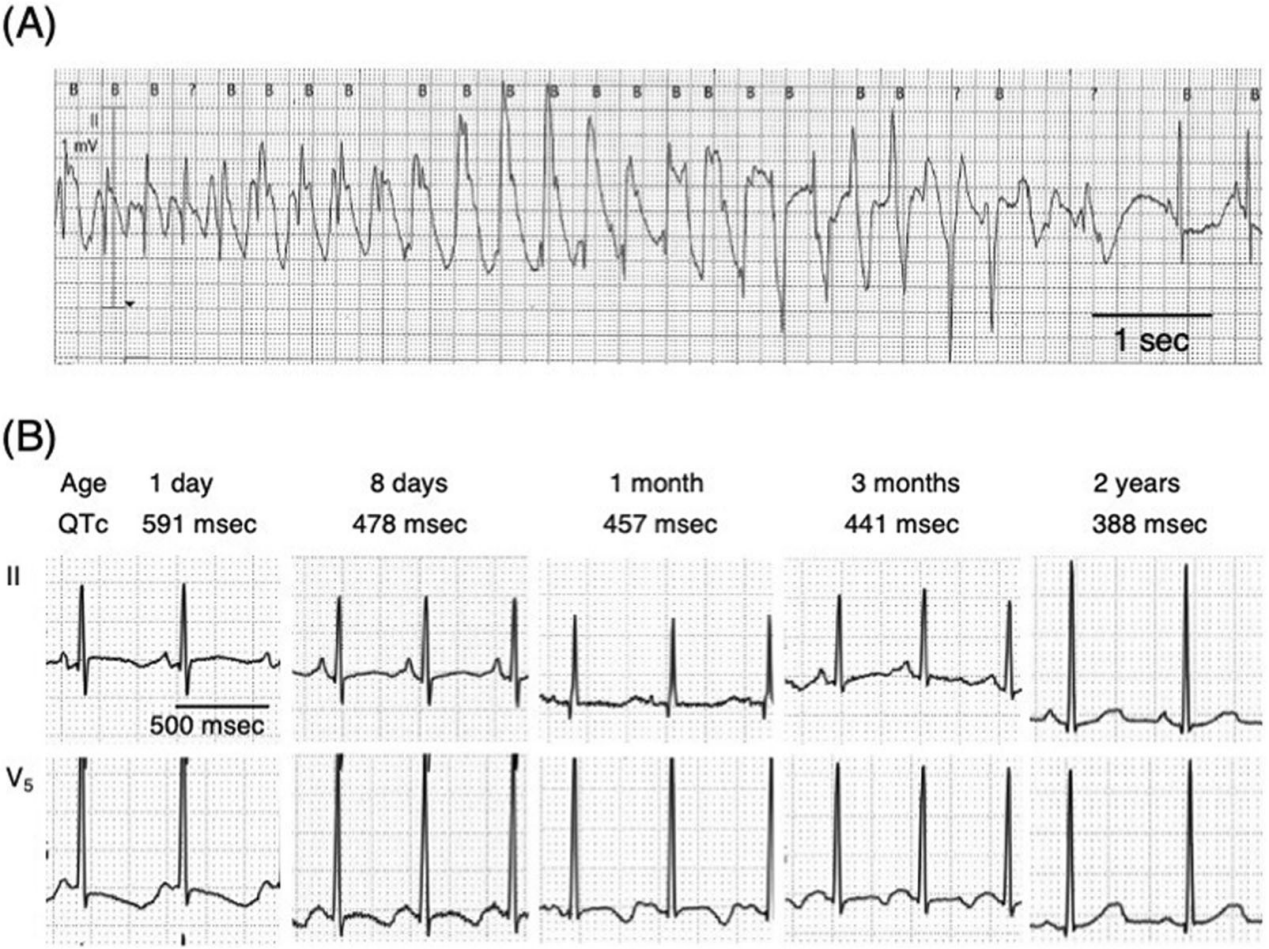
Postnatal electrocardiogram (ECG). **A** monitor ECG at 3 h after birth showing torsades de pointes. **B** Serial ECG showing that anti-arrhythmic drug therapy shortened the corrected QT interval (QTc) according to the Fridericia formula.

The patient was started on a regimen of magnesium sulfate and landiolol, which eliminated the torsade de pointes. Thereafter, treatment with mexiletine and propranolol gradually shortened the QT interval and effectively prevented ventricular tachycardia (Fig. 2B). The patient is still on medical therapy at 2 years of age and remains free of cardiac events. Neurological examinations revealed muscle hypotonia. At 3 months of age, the patient developed epileptic spasms. The interictal electroencephalography (EEG) results indicated hypsarrhythmia. The epileptic seizures were refractory to anti-seizure medication. Psychomotor development was severely delayed with no head control, eye pursuit, or speech sounds. The patient was fed via a gastrostomy tube due to feeding problems. Brain magnetic resonance imaging scans performed at 5 months of age showed no abnormal findings (Supplementary Fig. 1). These clinical features including hypotonia, seizures, and profound developmental delay were compatible with those of NDHSAL.

To reveal the underlying genetic etiology, the genomic DNA of the patient and the patient’s parents was extracted from the peripheral blood after obtaining written informed consent. This study was approved by the Asahikawa Medical University Research Ethics Committee (approval number 17112). Genetic testing for the known LQTS-associated genes identified no pathogenic variants in this patient^7^. Next, whole exome sequencing and variant filtering analysis were performed on the genes of the patient-parent trio, which identified a de novo heterozygous missense variant in *HECW2*, NM_001348768.2:c.4343 T > C,p.(Leu1448Ser). This missense variant was located in the C-terminal HECT domain of HECW2 and has been reported in another patient with NDHSAL in ClinVar (VCV001320205.1). A different missense change at the same codon (p.Leu1448Trp) has also been reported as likely pathogenic (ClinVar ID: VCV000801845.1)^8^. This missense variant was absent from the controls in public databases, such as the Exome Variant Server (http://evs.gs.washington.edu/EVS/), dbSNP (http://www.ncbi.nlm.nih.gov/snp/), and Genome Aggregation Database (gnomAD, https://gnomad.broadinstitute.org/). It was predicted to be damaged by in silico tools including SIFT, PolyPhen-2, and CADD (score 29.6). According to the American College of Medical Genetics and Genomics guidelines^9^, the variant was classified as pathogenic (PS2, PM1, PM2, PM5, PP3, and PP4).

In the present case, LQTS was diagnosed based on a prolonged QT interval and history of torsade de pointes. The patient harbored no pathogenic variants in the known LQTS-associated genes, but a pathogenic variant in *HECW2*. Cardiac comorbidities were reported in four cases of *HECW2*-related disorders (Supplementary Table 1). The first reported case of *HECW2*-related disorder with LQTS had prenatal-onset cardiomyopathy and harbored the heterozygous missense variant in *EIF2B2*, p.(Gly200Val), in addition to *HECW2* variant, NM_001348768.2:c.4334 A > G,p.(Glu1445Gly)^5^. Recessive variants in *EIF2B2* cause early onset vanishing white matter disease; however, heterozygous carriers of *EIF2B2* variants are asymptomatic^10^. Thus, the cardiac phenotype of the patient was considered to be due to *HECW2* variant. This is the second case that considers LQTS as a possible phenotype of a *HECW2* variant. Ubiquitylation has been shown to be implicated in the regulation of cardiac ion channels^11,12^. HECW2 is a member of the neuronal precursor cell-expressed developmentally down-regulated protein 4 (Nedd4) family of E3 ubiquitin ligases, which comprises nine members, including Nedd4-2. In particular, Nedd4-2 has been shown to regulate cell surface expression of cardiac voltage-gated sodium channel Na_v_1.5 through post-translational modification^11^. Nedd4-2 deficiency increases the cell surface expression of Na_v_1.5, resulting in cardiac proarrhythmic changes such as a prolonged QT interval^12^. LQTS patients with gain-of-function mutations of *SCN5A*, the gene encoding Na_v_1.5, have been reported to benefit from sodium channel blockers^13^. In the present case with *HECW2* variant, the use of mexiletine, a voltage-gated sodium channel blocker, in combination with a beta-blocker was effective in shortening the QT interval and suppressing cardiac events. Both HECW2 and Nedd4-2 are highly expressed in the heart tissue. Thus, future studies are required to elucidate the cardiac targets of HECW2. In conclusion, this study reports an additional case of a *HECW2*-related disorder with cardiac involvement and provides further evidence that the clinical manifestations of this disorder are not confined to the central nervous system.

## HGV Database

The relevant data from this Data Report are hosted at the Human Genome Variation Database at 10.6084/m9.figshare.hgv.3305.

### Supplementary information

Supplementary Table 1

Supplementary Figure 1

Supplementary figure legend
