# Supplementary figures and images for: A novel HECW2 variant in an infant with congenital long QT syndrome
Source: Hum Genome Var. 2023 Jun 6;10:17. doi: 10.1038/s41439-023-00245-w (PMC10244414; doi:10.1038/s41439-023-00245-w)

## Slide 1
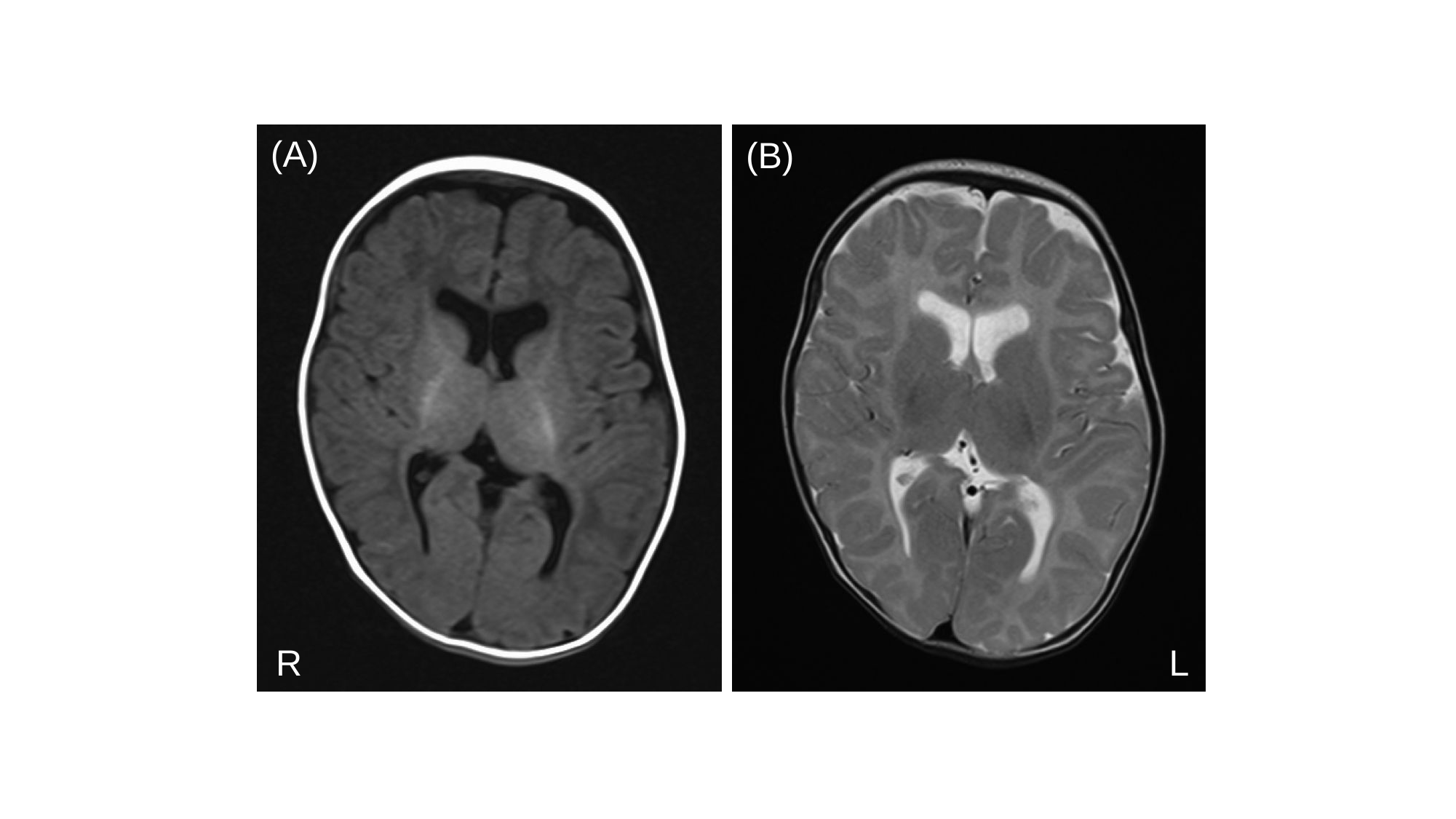

(A)
(B)
R
L

Supplement: Supplementary file 2 — Supplementary Figure 1 [file 41439_2023_245_MOESM2_ESM.pptx]
