# Supplementary material for: A novel HECW2 variant in an infant with congenital long QT syndrome
Source: Hum Genome Var. 2023 Jun 6;10:17. doi: 10.1038/s41439-023-00245-w (PMC10244414; doi:10.1038/s41439-023-00245-w)
Supplement: Supplementary file 3 — Supplementary figure legend [file 41439_2023_245_MOESM3_ESM.docx]

**Supplemental information**

Supplementary Figure 1. Brain magnetic resonance imaging scans of the patient with *HECW2*-related disorder. Axial T1-weighted (A) and T2-weighted (B) images obtained at the age of 5 months (3 months of corrected age) showed no abnormal findings.
